# Vocal foragers and silent crowds: context-dependent vocal variation in Northeast Atlantic long-finned pilot whales

**DOI:** 10.1007/s00265-017-2397-y

**Published:** 2017-11-06

**Authors:** Fleur Visser, Annebelle C.M. Kok, Machiel G. Oudejans, Lindesay A.S. Scott-Hayward, Stacy L. DeRuiter, Ana C. Alves, Ricardo N. Antunes, Saana Isojunno, Graham J. Pierce, Hans Slabbekoorn, Jef Huisman, Patrick J. O. Miller

**Affiliations:** 1 Behavioural Biology, Institute of Biology, Leiden University, Leiden, The Netherlands; 2 Institute for Biodiversity and Ecosystem Dynamics, University of Amsterdam, Amsterdam, The Netherlands; 3 Kelp Marine Research, Hoorn, The Netherlands; 4 Centre for Research into Ecological and Environmental Modelling (CREEM), University of St Andrews, Scotland, UK; 5 Department of Mathematics and Statistics, Calvin College, Grand Rapids, MI USA; 6 Sea Mammal Research Unit, Scottish Oceans Institute, University of St Andrews, Scotland, UK; 7 Ocean Giants Program, Wildlife Conservation Society, New York, NY USA; 8 Oceanlab, University of Aberdeen, Aberdeenshire, UK; 9 CESAM and Departamento de Biologia, Universidade de Aveiro, Aveiro, Portugal; 10 CSIC, Vigo, Spain

**Keywords:** Animal communication, Social behaviour, Pilot whale, Call, Foraging, Acoustic tags

## Abstract

**Abstract:**

Vocalisations form a key component of the social interactions and foraging behaviour of toothed whales. We investigated changes in calling and echolocation behaviour of long-finned pilot whales between foraging and non-foraging periods, by combining acoustic recordings and diving depth data from tagged individuals with concurrent surface observations on social behaviour of their group. The pilot whales showed marked vocal variation, specific to foraging and social context. During periods of foraging, pilot whales showed more vocal activity than during non-foraging periods (rest, travel). In addition to the expected increase in echolocation activity, call rates also increased, suggesting that pilot whales communicate more during foraging. Furthermore, calls with multiple inflections occurred more often immediately before and after foraging dives and during the early descent and late ascent phases of foraging dives. However, these calls were almost never detected at diving depths of the tagged whale beyond 350 m. Calls with no or few inflections were produced at all times, irrespective of diving depth of the tagged whale. We discuss possible explanations for the distinct vocal variation associated with foraging periods. In addition, during non-foraging periods, the pilot whales were found to be more silent (no calling or echolocation) in larger, more closely spaced groups. This indicates that increased levels of social cohesion may release the need to stay in touch acoustically.

**Significance statement:**

Social toothed whales rely on vocalisations to find prey and interact with conspecifics. Species are often highly vocal and can have elaborate call repertoires. However, it often remains unclear how their repertoire use correlates to specific social and behavioural contexts, which is vital to understand toothed whale foraging strategies and sociality. Combining on-animal tag recordings of diving and acoustic behaviour with observations of social behaviour, we found that pilot whales produce more calls during foraging than during non-foraging periods. Moreover, highly inflected calls were closely associated to the periods around and during foraging dives. This indicates enhanced communication during foraging, which may, for example, enable relocation of conspecifics or sharing of information. Whales reduced their vocal activity (calling and echolocation) at increased levels of social cohesion, indicating that in certain behavioural contexts, closer association (i.e. more closely spaced) may release the need to stay in touch acoustically.

**Electronic supplementary material:**

The online version of this article (10.1007/s00265-017-2397-y) contains supplementary material, which is available to authorized users.

## Introduction

Vocalisations play an important role in the life of social animals (e.g. Simmons et al. 2003; Marler and Slabbekoorn 2004; Owings and Morton 2006; Liebal et al. 2013). Communicative functions that may directly affect individual survival and reproductive success are typically reflected in context-dependent vocalisation rates and types. Call rates, for example, often increase in response to perceived predation risk or when animals aggregate for feeding, spawning or migration. Moreover, most animals have a repertoire from which they select specific vocal variants depending on the context: feeding calls differ from alarm calls, advertisement songs for long-range communication are distinct from close range aggressive chatters and specific calls are used in the signalling of individual and group identity. Vocal communication is of particular importance for cetaceans, as their marine environment often constrains the use of visual cues, while sounds can be detected over long distances (Payne and Webb 1971; Tyack 2000; Nummela et al. 2004; Miller 2006).

Toothed whales use echolocation clicks to localise and capture prey during foraging (e.g. Miller et al. 2004; Madsen et al. 2013). Moreover, most species are highly social and produce a large variety of communicative vocalisations (calls), which may function to maintain group cohesion or coordinate activities, or in the recognition of individuals or group members (Janik and Slater 1998; Yurk et al. 2002; Gero et al. 2016). Nonetheless, apart from these broad functions, it often remains unclear if and how vocal variation correlates to more specific social and behavioural contexts. This is partly due to limitations inherent in studying wild, deep-diving whales that perform a large part of their behaviour out of sight of human observers and occur in groups or larger aggregations in which multiple animals vocalise at the same time. This challenges the ability to correlate vocalisation patterns with direct observations of behaviour and to link the source of a call to producers and potential receivers. However, information about context-dependent fluctuations in vocal activity and repertoire use is vital to understand toothed whale foraging strategies and sociality.

Long-finned pilot whales (*Globicephala melas*) are highly social cetaceans that live in long-term stable, matrilineal groups (Amos 1993; Ottensmeyer and Whitehead 2003; de Stephanis et al. 2008). They occur mostly in tightly spaced, behaviourally coordinated groups of about 10 individuals, within larger aggregations (Cañadas and Sagarminaga 2000; Senigaglia and Whitehead 2012; Visser et al. 2014). The species has a complex vocal repertoire, consisting of broadband echolocation clicks and a wide variety of call types. Calls, narrowband, frequency-modulated vocalisations that may also contain click series, vary along an apparent continuum from simple to highly complex types (Taruski 1979; Nemiroff and Whitehead 2009; Sayigh et al. 2013 and Marrero Pérez et al. 2017 for short-finned pilot whales (*G. macrorhynchus*)). This variation may be functionally important if the use of particular call variants is correlated with specific social, behavioural or environmental contexts. Both long- and short-finned pilot whales produce repeated call types, e.g. rhythmic sequences, thought to function in social cohesion or identification (Sayigh et al. 2013, Zwamborn and Whitehead 2017).

Long-finned pilot whales perform deep foraging dives, up to 800 m depth, during foraging periods typically consisting of series of deep dives and intermittent shallow dives (Baird et al. 2002; Sivle et al. 2012; Visser et al. 2014, 2016). Although the number of observations is still limited, it seems that they employ a social foraging strategy, whereby group members to a large degree synchronise the timing of their foraging periods, although they do not necessarily synchronise individual dives (Aoki et al. 2013; Visser et al. 2014). During foraging, surface group size is reduced and surfacing individuals become more widely spaced (Visser et al. 2014). Data on whether deep-diving toothed whales forage cooperatively at depth is limited and, thus far, cooperative foraging has not been reported. However, they do produce social calls during foraging, which may function to maintain or re-establish bonds with group members performing deep foraging dives individually or in small groups (Whitehead 1989; Aguilar de Soto et al. 2011; Jensen et al. 2011; Marrero Pérez et al. 2017). Hence, long-finned pilot whales that are closely associated at the surface while resting or traveling, can temporarily become spaced hundreds of metres apart during foraging, and may consequently become more reliant on vocalisations to transfer information, or relocate group members (Jensen et al. 2011; Visser et al. 2014).

To gain further insight into the function of vocalisations for social toothed whales that rely on sound to find prey and mediate social interactions, we combined on-animal tag data of diving patterns, group vocal activity and repertoire use with surface observations of social behaviour and group size. Under the hypothesis that pilot whales will alter characteristics of their communication as a function of social and behavioural context, we investigated (1) whether the occurrence of specific long-finned pilot whale vocalisation types was associated with foraging periods and diving phase, and (2) whether their calling activity and the use of specific vocalisations was correlated to the degree of surface social cohesion, as quantified by animal numbers and proximity to group members and other groups.

## Methods

The behaviour of long-finned pilot whales (*Globicephala melas*) was visually monitored from the research vessel M/S Strønstad (29 m) in the Vestfjord basin off Lofoten, Norway (66°–70° N latitude), a coastal and relatively shallow (100–600 m) fjord, in May/June of 2008, 2009 and 2010 (Kvadsheim et al. 2009). We used multi-sensor tags to record the diving behaviour and acoustic scene of individual pilot whales. These data can be used to identify behavioural patterns (e.g. foraging; Madsen et al. 2013) of tagged individuals. An individual was tagged at the first available opportunity using a suction cup-attached archival tag (DTAG, Johnson and Tyack 2003). Concurrent with the tag recordings, we recorded group-level surface behavioural parameters of the tagged whale’s group.

### Tag recordings

Patterns in vocal behaviour were analysed using the stereo sound recordings on the DTAGs (16-bit resolution at 96 or 192 kHz sampling rate). The start and end time of all vocalisations recorded on the tag that were audible and visible on the spectrograms were marked by two independent observers using Adobe Audition 2.0 (Blackman-Harris window, 4096 sample FFT, 75% overlap, 180 dB dynamic range). In order to analyse sounds that were likely most relevant to the tagged whale’s acoustic scene, all sounds were classified by perceived signal-to-noise ratio (SNR) as (1) faint, barely detectable, often only partly visible vocalisations and (2) loud, clear and complete vocalisations (following Alves et al. 2014; Visser et al. 2016). Only vocalisations in class (2) were included in analysis.

**Fig. 1:**
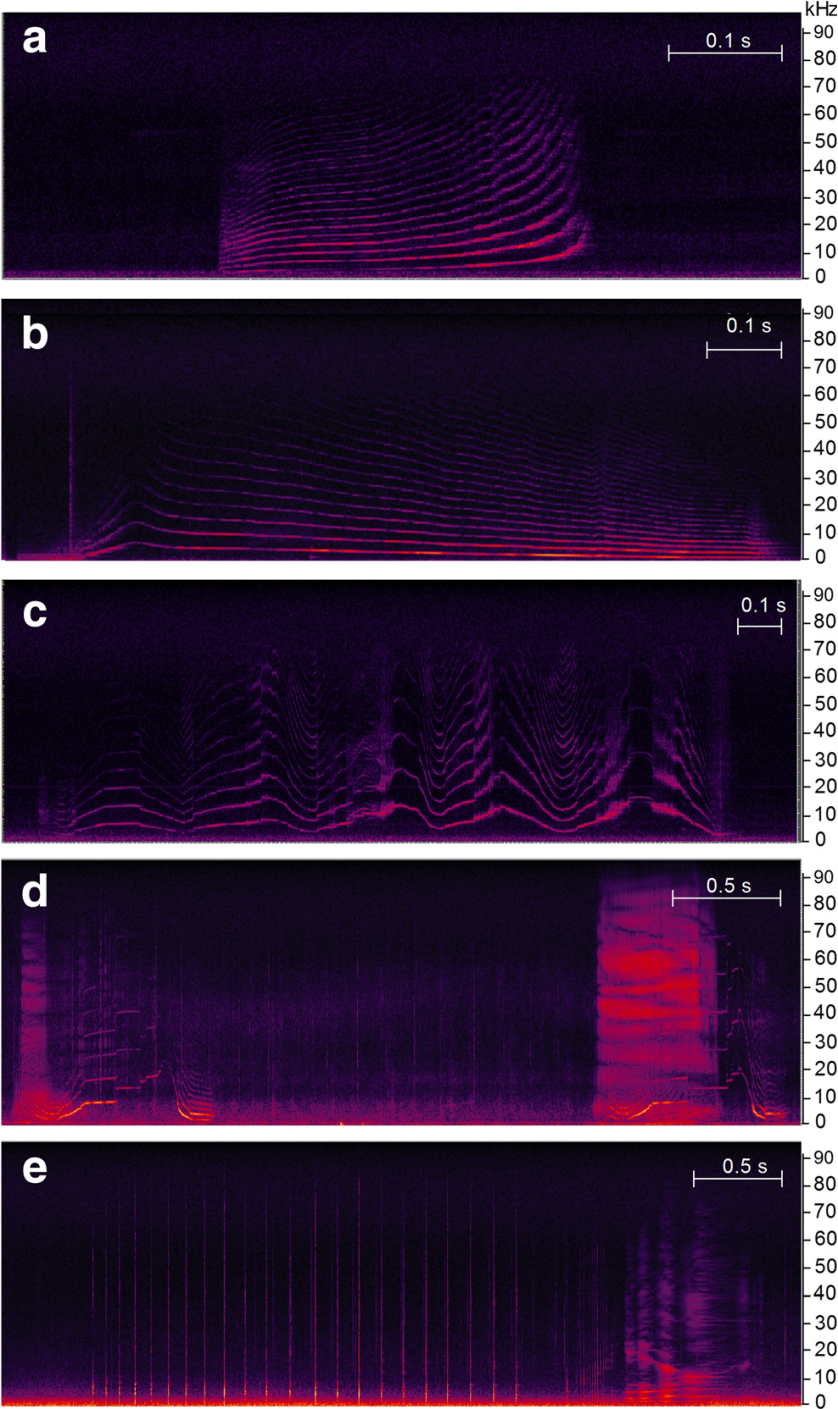
Spectrogram examples of call types and click series. **a** Non-inflected call (no inflections). **b** Inflected call with 1 inflection. **c** Highly inflected call with > 2 inflections. **d** Two inflected calls (2 inflections) with click series component. **e** Click series. Slow click series with individually distinguishable clicks (click train) followed by fast click series (buzz), made in a foraging context as part of the prey search and capture attempt phases of bio-sonar-based foraging. Spectrogram settings: Blackman-Harris window, 4096 sample FFT, 75% overlap, 132 dB dynamic range

To investigate vocal variation between different behavioural contexts, we identified the occurrence of calls, clicks and silent periods. Calls (often termed ‘whistles’, see Madsen et al. 2012) likely function as communication signals. They were identified by narrowband, often harmonic bands in the spectrogram and classified into three categories as (a) non-inflected calls (no frequency inflections), (b) inflected calls (1–2 inflections) or (c) highly inflected calls (more than 2 inflections; Figs. 1a–d, S1), based on earlier classifications by Taruski (1979) and Weilgart and Whitehead (1990). An inflection was defined as a shift from increasing to decreasing frequency, or vice versa, visible in the fixed visualisation settings of the spectrogram (0–20 kHz, 8 s window). We thus classified calls into a limited number of categories, and focus on limited vs. abundant use of inflections. This broad classification was applied as the long-finned pilot whale vocal repertoire may have many more vocal variants, which may vary along continuous scales dependent on behavioural state (Taruski 1979; Sayigh et al. 2013 and Marrero Pérez et al. 2017 for short-finned pilot whales). Our use of a restricted number of broad tonal call categories avoided categorisation problems of a multitude of gradually changing, more or less overlapping call variants. Calls could also include a click series component (Sayigh et al. 2013; Zwamborn and Whitehead 2017; Fig. 1d). These calls were classified in the same way as calls without a click series component, using the number of frequency inflections of the harmonic band. Calls that could not be individually classified (< 0.2 s apart or overlapping) were classified as a composite call (e.g. non-inflected + inflected call). We did not distinguish between calls produced by the tagged whale and vocalising conspecifics as there is no method available that would be able to perform this distinction with sufficient accuracy for our dataset. Echolocation click series, thought to mainly function to locate and catch prey (e.g. Madsen et al. 2013), were defined as series of consecutive, short broadband clicks, less than 2 s apart (Fig. 1e). Given the aim to investigate differences in vocal behaviour between foraging and non-foraging contexts, this vocalisation type was also included in the analysis of vocal behaviour. To define periods of silence (no calling and no clicking), opposed to naturally occurring pauses between calls and/or echolocation vocalisations, we conducted a log-frequency analysis of vocalisation intervals. This defined the break point between pauses and silent periods at 24.5 s. Silence was defined as a period with an interval > 24.5 s between consecutive vocalisations in class 2 (Visser et al. 2016).

Diving depth of the tagged whales was obtained by calibrated conversion of the pressure recorded on the DTAGs at 50 Hz, down sampled to 5 Hz. Each diving record was classified into periods of foraging (associated with dives > 34 m; Baird et al. 2002) and non-foraging periods. Using the definition in Visser et al. (2014), a foraging bout started at the first dive deeper than 34 m and included all following dives deeper than 34 m until no other dive deeper than 34 m was initiated within 14.5 min after the end of the last dive. Hence, a foraging period could hold one to several foraging dives and intermittent/post dive near surface behaviour. During all dives exceeding 34 m, echolocation-based foraging sounds (buzzes) were recorded in the acoustic scene of the tagged whale, indicating that these deeper dives were foraging dives (Visser et al. 2014). Group members in the studied population to a large degree synchronise the timing of their foraging periods (Visser et al. 2014). Therefore, the behavioural state of the tagged whale (i.e. foraging or non-foraging) was considered to be representative for the behavioural state of its group.

### Visual data collection

Visual focal follow observations of group-level behavioural metrics were made from the observation platform at 6 m above water level. The research vessel aimed to maintain a distance of 100–400 m from the focal group. Tracking was aided by the radio beacon on the tags, informing observers when the tagged whale surfaced. Behavioural data were collected by two dedicated observers, alternating in 6-h shifts, assisted by a second person recording the data. The distance estimates of the two observers were calibrated by a laser range finder, and by comparison of their estimates of a range of distances to a GPS-equipped buoy. Sampling was conducted during all hours of the day, as the high latitude provided 24-h daylight conditions.

The focal follow observation protocol and group definition is detailed in Visser et al. (2014) and summarised here. A focal group was defined as the group of individuals in closer proximity to the tagged whale and each other than to other individuals in the area. To assess focal group membership, we first defined different spacing categories based on the distances between individuals (in body lengths; categories: very tight (< 1 body lengths (BL)), tight (1–3 BL), loose (3–15 BL), very loose (> 15 BL) and solitary (no other individual in focal area and/or at a larger spacing category from its nearest neighbour than the nearest neighbour from its own nearest neighbour)). When the tagged individual surfaced, the first step in estimating group size was to determine its nearest neighbour. The focal group included all individuals with similar proximity (according to the individual spacing categories) to the tagged individual or other group members as the nearest neighbour. Thus, focal group membership was based on the relative distribution of the individuals around the tagged whale (Visser et al. 2014). Thereby, the focal group represents a set of individuals that have a similar chosen distance to their nearest neighbour (Krause and Ruxton 2002) at a certain point in time; it does not necessarily represent a stable social unit. In order to enable quantification of the presence and proximity of other individuals in relative proximity to the focal whale, we also counted the number of individuals and groups in the focal area, defined as the 200 m radius around the tagged individual. We scored 4 group-level parameters, using scan sampling, to quantify the number of animals near the tagged whale and the distance between individuals and groups: (1) group size, (2) number of individuals in the focal area, (3) distance to the nearest other group (categories: < 50 m, 50–100 m, 100–200 m, 200–500 m, 500–1000 m, none in sight) and (4) individual spacing (categories; defined above). Individuals were classified as associated with a calf when an adult-sized animal was (very) tightly paired with a calf during the majority of its surfacings. Non-focal groups were defined in a similar way as the focal group, as all individuals in closer proximity to each other than to other individuals in the area. Group-level behaviour was always sampled when the tagged individual was visible at the surface. Data were recorded at 2-min intervals, or at first surfacing of the tagged individual following dives longer than 2 min (first available opportunity for visual recording of group-level behaviour at the surface). The 2-min resolution was used as a sampling interval as it is a time interval that is shorter than the interval at which transitions in behaviour were expected to occur, while allowing for sufficient time for observers to collect high-quality data for each record. It was not possible to record data blind because our study involved field observations of free-ranging focal animals.

### Statistical analysis of vocalisation patterns

We investigated whether vocalisation patterns varied as a function of group-level behaviour and diving state (foraging or non-foraging) using Generalised Estimating Equations (GEEs; Hardin and Hilbe 2003). The input data comprised time series from 7 tagged focal animals and their focal groups. We included all data recorded from 30 min after the tagging vessel left the tagged animal up to the release of the tag or up to the start of sound exposure experiments (reported in Miller et al. 2012). For each sampling interval of group-level behaviour (time bin), we determined (a) whether the interval occurred during a deep diving period (foraging) or a non-foraging period (i.e. classified as ‘deep diving period’ if > 50% of the duration of a sampling record overlapped with a deep diving period), and (b) the proportion of time occupied by each vocalisation type (all calls, clicks, silence). This enabled matching of group-level surface behaviour occurring during a 2-min record with the vocal behaviour recorded in those same 2 min, as a function of being in a foraging period or in a non-foraging period.

First, each vocalisation type was modelled as a binomial response variable against *diving state* (foraging or non-foraging) and the four covariates for *group-level behaviour*, plus all two-way interaction terms between diving state and the other covariates. The duration of the response variable (e.g. duration of silence in a time bin) was modelled as the “successes” and the duration of the time bin as the “trials” of a binomial distribution. Interaction terms were included to investigate whether a relationship between vocal and group-level behaviour varied between periods of foraging and non-foraging behaviour. Individual observations were weighted by bin length to control for sampling intervals differing in duration (due to variation in surfacing intervals of the tagged whales). To account for our repeated measures design, we specified a blocking unit (focal follow ID) in the GEE, so that residuals were permitted to be correlated within, but assumed to be independent between focal follows. Models were fitted using the robust variance estimator (Zorn 2006) and an independent working correlation structure. Model selection was performed using a hypothesis-based backwards ANOVA (sequential Wald test). After running the binomial GEE model and the ANOVA for each vocalisation type, the term with the largest *p* value in the ANOVA model was removed, and the GEE model was rerun, until all retained terms in the ANOVA were significant (*P* < 0.05). Analyses were performed using the package ‘geepack’ (Højsgaard et al. 2006) in R version 2.14.1 (R Development Core Team 2011).

Second, we used a multinomial GEE to investigate the highest occurring level of inflections in relation to group-level and diving behaviour. This analysis required equal-length time bins and the dataset was resampled to 2-min bins. The categorical ordinal response was the most inflected call type (no call, non-inflected, inflected or highly inflected) occurring within each 2-min bin. It was modelled against the same set of covariates and two-way interaction terms as the binomial GEE analysis, using focal follow ID as blocking unit. Model selection was achieved as before, using Wald statistic *p* values and backwards selection. The best model was chosen using QIC_u_ (Quasilikelihood Information Criterion under the independence model; Pan 2001). Models were fitted using SAS software, v9.4.

To describe estimated relationships between the response variable and the selected covariates with the multinomial GEE, predictions were made to all levels of a single covariate, while fixing the other covariates in the model at the most prevalent level (factors) or the median (continuous covariates). For example, to describe relations between vocalisation types and group size, values for the other 3 group-level covariates were fixed at their median or most prevalent level (e.g. ‘tight’ for individual spacing). Predominant or median covariate values were used to ensure model predictions represented a valid combination in the dataset. Bootstrap percentile-based 95% confidence intervals were estimated using robust standard errors.

## Results

We recorded the individual diving behaviour, acoustic scene and associated group-level surface behaviour of 7 tagged individuals (focal follow duration 32.1 h; *N* = 556 behavioural samples; 14 foraging and 17 non-foraging periods). All seven focal groups holding the tagged individuals were part of larger aggregations of 60–100 pilot whales, generally composed of several groups spread out over a larger area. The tagged individuals were two medium-sized individuals associated with calves, three medium-sized individuals without a calf and two large adults (sizes were field estimates; Table 1). Focal group size ranged from 1 to 30 individuals, with a median of 11 (interquartile range (IQR): 6). Group size was not related to spacing between individuals. All tagged whales performed both foraging and non-foraging dives, except for one individual that performed only non-foraging dives. Periods of foraging occurred during 36% of the time. Non-foraging behaviour typically consisted of restful or traveling behaviour, with limited observation of surface socialising.

**Table 1:** Summary of focal follows. TAG ID = identification number of tagged whale. Individual class: L = large adult, M = medium-sized adult, C = consistently associated with calf. Max. depth = maximum diving depth in tag record. Infl. = inflected

TAG ID	Date	Ind. class	Follow duration (h)	Group size (range)	Max. depth (m)	N calls/min Non infl./Infl./Highly infl.	No. foraging/non-foraging periods
gm09_137a	17-05-2009	MC	6.2	6–16	291	2.8/1.2/0.4	2/3
gm09_138a	18-05-2009	M	2.1	7–30	408	3.7/2.3/0.5	1/1
gm09_156b	05-06-2009	L	4.1	1–30	554	1.5/0.8/0.1	2/2
gm10_143a	23-05-2010	L	8.2	1–11	492	2.6/1.1/0.2	4/5
gm10_152b	01-06-2010	M	0.8	12	74	7.6/4.5/0.6	1/1
gm10_157b	06-06-2010	MC	8.9	1–30	617	2.4/1.7/0.6	4/4
gm10_158d	07-06-2010	M	1.8	6–10	21	0.8/0.4/0.01	0/1

Pilot whale vocalisations were recorded during 40% of the time (Table 2). Click series comprised the predominant part of the time spent vocalising. Of the call types, non-inflected calls were produced most often. The highly inflected calls were relatively rare. Silent periods ranged from 24.6 s to 39.2 min in duration, covering 39% of the time (Table 2). Comparison of the vocal behaviour between foraging and non-foraging behaviour and between group-level behaviours revealed distinct patterns in the occurrence of the different call types and silent periods, which were confirmed by the GEE analyses (Table 3).

**Table 2:** Number, duration and percentage of time recorded for each vocalisation type, pauses and silent periods, and vocalisation rate during foraging (F) and non-foraging (NF) diving periods (total recording time 32.1 h). Click series and silent periods: vocalisation rate = average % of time recorded per individual; Calls: vocalisation rate = average no. calls per minute per individual

Vocalisation type	No.	Mean duration (SD) (s)	% of time	Vocalisation rate in F/NF diving period (±SEM) (ratio F/NF)
Total vocalisations	10,393		40.2	
- Non-inflected calls (no inflections)	4929^a^	0.79 (1.1)	3.1^a^	4.2 (0.7)/1.8 (0.4) (2.3)^a^
- Inflected calls (1–2 inflections)	2643^a^	0.95 (1.4)	1.9^a^	2.3 (0.5)/1.0 (0.2) (2.3)^a^
- Highly inflected calls (> 2 inflections)	676^a^	1.23 (0.7)	0.7^a^	0.5 (0.16)/0.2 (0.09) (2.4)^a^
- Click series	3630	15.3 (37.3)	39.1	57.0 (4.9)/24.0 (4.7) (2.4)
Pauses^b^	4980	4.6 (4.6)	20.8	
Silent periods^b^	430	106.1 (195.6)	39.0	16.7 (6.2)/54.9 (5.7) (0.3)

^a^Including single and composite vocalisations

^b^Pauses were < 24.5 s; silent periods were > 24.5 s

**Table 3:** Wald statistic *p* values for retained covariates (*P* < 0.05) in the three binomial and multinomial GEE-based vocalisation models (binomial: silence and calls; multinomial: call inflections). Empty cell: parameter not retained best model. Parameter estimates and standard errors given in Tables S1 and S2

	Vocalisation type		
Covariate	Silence	Calls	Call inflections
Diving state (foraging/non-foraging)	< 0.0001	0.005	0.04
Group size	0.13^a^		
Distance to other group			0.34^a^
No. individuals in focal area	0.21^a^		
Individual spacing	< 0.0001	0.01	0.006
Diving state/group size	< 0.0001		
Diving state/distance to other group			< 0.0001
Diving state/no. individuals in focal area	< 0.0001		
Diving state/individual spacing	< 0.0001	< 0.0001	< 0.0001

^a^Parameter retained in best model because included in significant 2-way interaction term

### Foraging vs. non-foraging behaviour

**Fig. 2:**
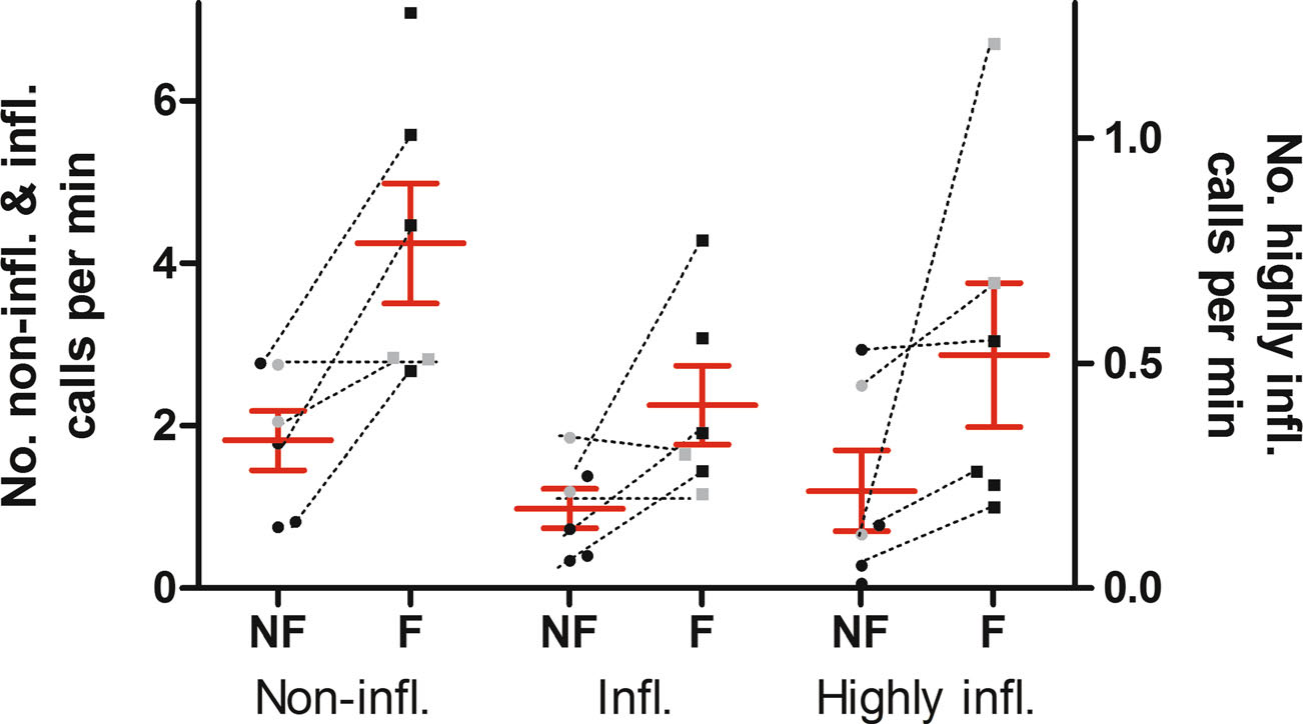
Non-inflected, inflected and highly inflected call rates in the acoustic scene of a tagged individual during non-foraging (NF) and foraging (F) periods (median ± SEM). Data points show call rates per individual tag record during non-foraging periods (filled circles) and foraging periods (filled squares). Dashed lines link call rates of the same tag record. Grey icons: adults associated with a calf; black icons: adult not associated with a calf. Infl. = inflected

Call rates and the level of inflections recorded were significantly higher during foraging periods than during non-foraging periods (Fig. 2). Congruently, the occurrence of silent periods was significantly reduced during foraging periods (*P* = 0.005, 0.04 and < 0.0001 respectively; Tables 2 and 3). Of the total of 145 highly inflected calls produced during foraging dives (> 34 m), 105 (72%) were recorded in dives with a maximum depth > 120 m (range 121–615 m). The remaining 40 calls were recorded during two dives to ~ 62 m of whale gm09_137a (two consecutive dives following a foraging dive to 291 m). Of all highly inflected calls recorded outside of foraging dives, 35% were produced within 15 min of the start or end of a foraging dive > 120 m (range 121–615 m), whereas 16% were produced within 15 min of the start or end time of a foraging dive < 75 m (range 40–73 m; Suppl. Fig. S2).

### Vocal production in the dive cycle

**Fig. 3:**
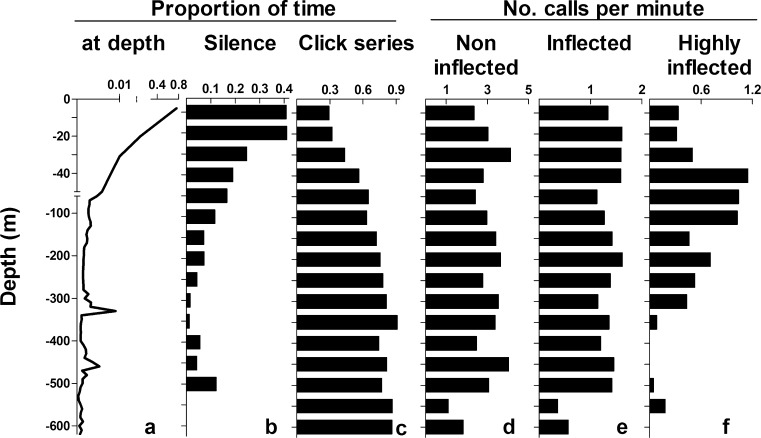
Time spent at depth and vocal activity as a function of diving depth. **a** Proportion of time spent at depth (aggregate of all tag records). **b**, **c** Proportion of time silence and click series were recorded. **d**–**f** Recorded call rates of non-inflected, inflected and highly inflected calls. Note scale differences on *y*-axis between 0–50 and 60–600 m. Maximum diving depth differed between individuals (Table 1) and values for larger depths represent fewer individuals (all: *N* = 7; > 350 m: *N* = 4)

**Fig. 4:**
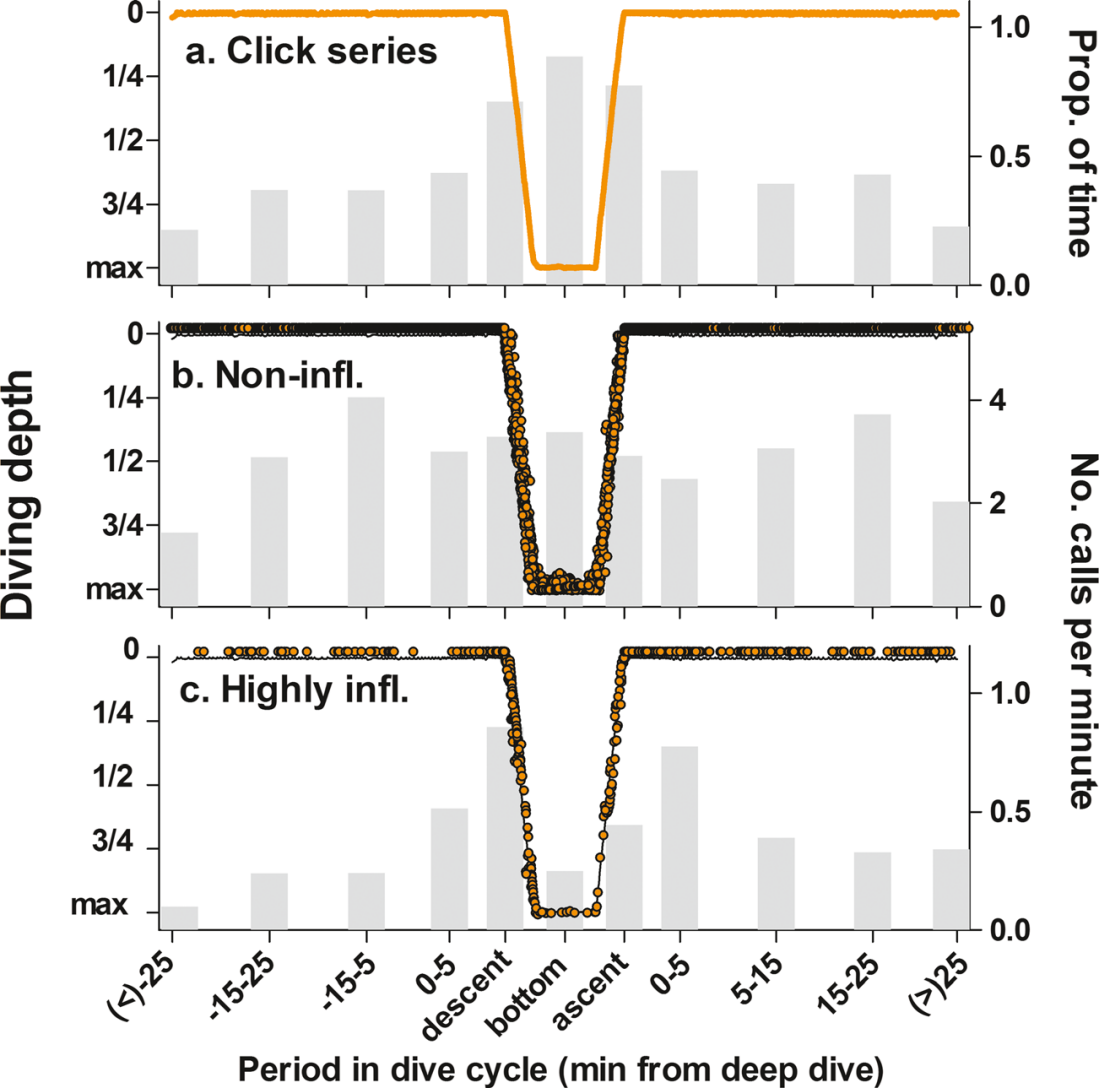
Timing and vocalisation rates of all **a** click series, **b** non-inflected calls and **c** highly inflected calls as a function of diving phase, relative to the timing of the nearest foraging dive. Infl. = inflected. Graphs show a typical dive of a long-finned pilot whale, preceded and followed by a 30-min period of near surface dives (black line). Vocalisations could occur prior to or following a foraging dive, or during the descent, bottom or ascent phase of a foraging dive. Descent phase: period from when the whale left the surface until the first time depth exceeded 90% of maximum dive depth. Ascent phase: period from the last time depth exceeded 90% of maximum dive depth until the whale reached the surface. Bottom phase: period between descent and ascent phase. Occurrence in quarterly sections of descent and ascent phases was determined from the call recording depth relative to the maximum dive depth of the foraging dive in which the call occurred. Orange line or symbols indicate the relative timing in the dive cycle of all clicks series, non-inflected and highly inflected calls recorded. Bars indicate vocal rates for each time bin before or after the nearest foraging dive, and during the three stages of the foraging dive

The seven tagged whales spent the majority of time (76%) in the upper 10 m of the water column. Less time was spent at greater depths, although small peaks reflected more time spent around 310 and 460 m deep than at adjacent depths (Fig. 3a). The occurrence of silent periods was highest in the top 20 m of the water column, steadily decreased with increasing depth, and became rare deeper than 100 m (Fig. 3a). This was largely explained by the depth distribution of occurrence of click series. This was lowest at shallow depths and steadily increased to near full-time occurrence of clicking at depths greater than 100 m (70–91% of the time; Fig. 3c). Increased occurrence of clicking at larger depths was confirmed by a strong increase in the proportion of time clicking in foraging dives, peaking during the bottom phase, compared to periods outside of foraging dives (Fig. 4a).

Depth distributions varied between call types. Recordings of non-inflected and inflected calls showed limited variation in relation to depth. In the two animals which dove > 550 m, occurrence of these calls was somewhat reduced at the deepest diving depths (Fig. 3d, e). In contrast, recordings of the highly inflected calls peaked between 31 and 100 m and were almost absent beyond 350 m depth (Fig. 3f; *N* = 4 animals diving > 350 m deep). While rarely produced at larger diving depths, recordings of highly inflected calls were clearly associated with foraging dives of the tagged whale (Fig. 4c). The rate of recording of highly inflected calls increased during the 5 min prior to a foraging dive (0.51 calls/min) rates and further increased during the first quarter of the descent phase (1.2 calls/min). Rates were then reduced throughout the remainder of the descent (0.55 calls/min), the bottom phase (0.25 calls/min) and the first half of the ascent phase (0.28 calls/min), after which increasingly more highly inflected calls were recorded during two final quarters of the ascent phase (0.44 and 0.61 calls/min, respectively) and during the first 5 min following the dive (0.59 calls/min). This pattern was not present for the less-inflected call types, which occurred throughout the dive cycle with limited variation in recording rates (non-inflected: Fig. 4b). In total, 50% of foraging dives contained highly inflected calls. Foraging dives with highly inflected calls contained (median (IQR, range)) 1 (3.3, 0 to 49) calls during the descent, 0 (0.3, 0 to 7) calls during the bottom phase and 0.5 (2, 0 to 14) highly inflected calls during the ascent phase, respectively.

### Social context of vocalisations

**Fig. 5:**
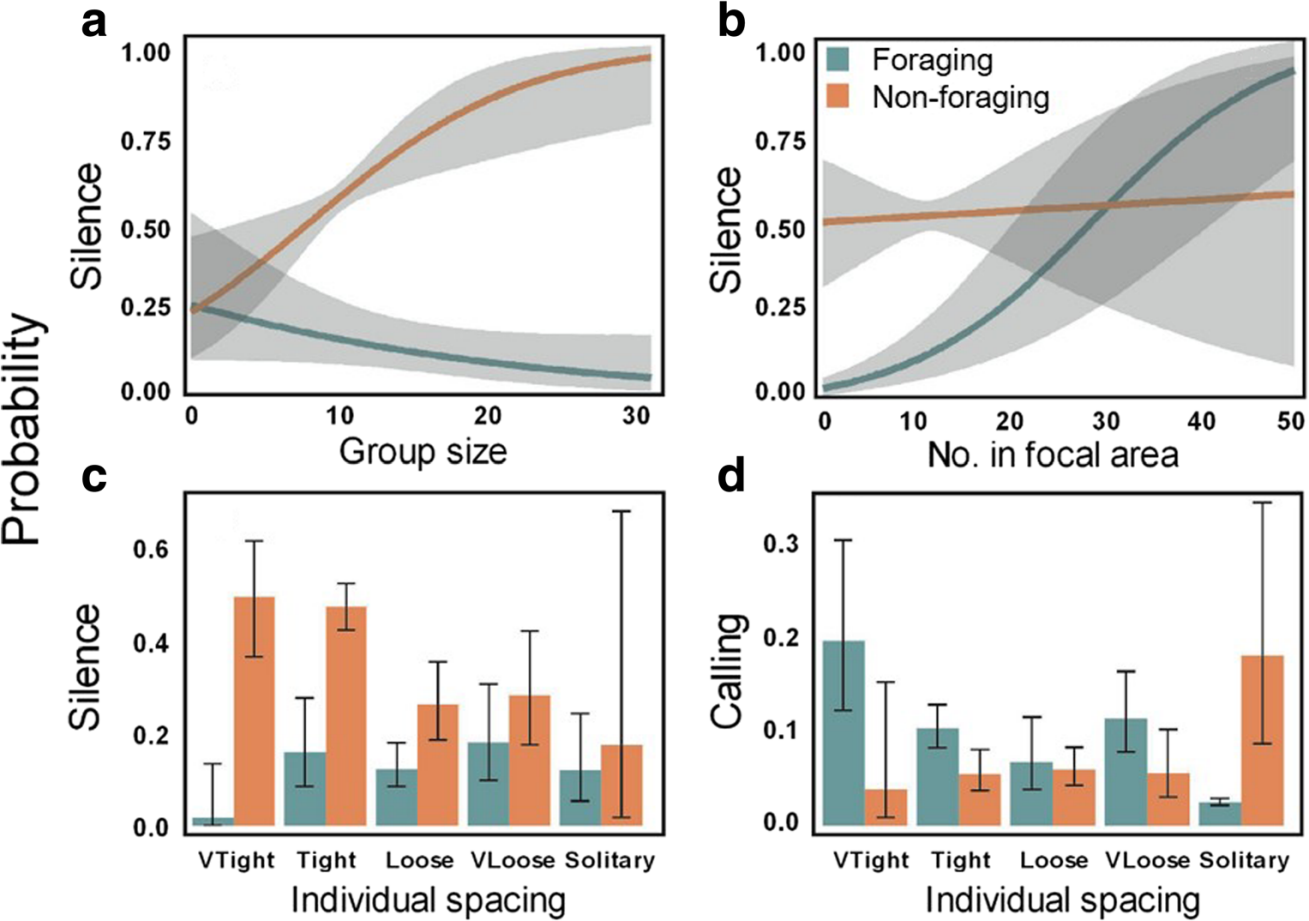
Binomial GEE model predictions for the significant relationships between vocal and group-level behaviour for foraging and non-foraging periods. **a** Group size. **b** No. in focal area. **c**, **d** Individual spacing. All relationships show contrasting patterns between foraging and non-foraging periods. Model predictions made to group size = 9; number in focal area = 15; tight individual spacing; 200–500 m to nearest other group and non-foraging period. Shaded areas (**a**, **b**) and error bars (**c**, **d**) represent 95% confidence intervals

Animal numbers and the spacing between individuals and groups significantly affected calling patterns and the occurrence of silent periods (GEE model results; Tables 3, S1, S2). Moreover, in both the best GEE models for silence and calls, the two-way interaction terms between diving state (foraging or non-foraging period) and group-level parameters were retained, indicating that the relationships between vocalisations and group behaviour were different (indeed they were often opposite), between foraging and non-foraging periods (Fig. 5).

Whales were more silent when they were organised in larger, or more tightly spaced groups (non-foraging periods; Fig. 5a, c; Table 3), or in the presence of a larger number of animals in the focal area (< 200 m; foraging periods; Fig. 5b). The occurrence of calling was related to the spacing between group members (Table 3). During foraging periods, calls were more often recorded in more tightly spaced groups, particularly when group members were separated < 1 body length, and rarely when individuals were solitary. An opposite trend (less calling when more closely spaced) was observed during non-foraging periods (Fig. 5d).

**Fig. 6:**
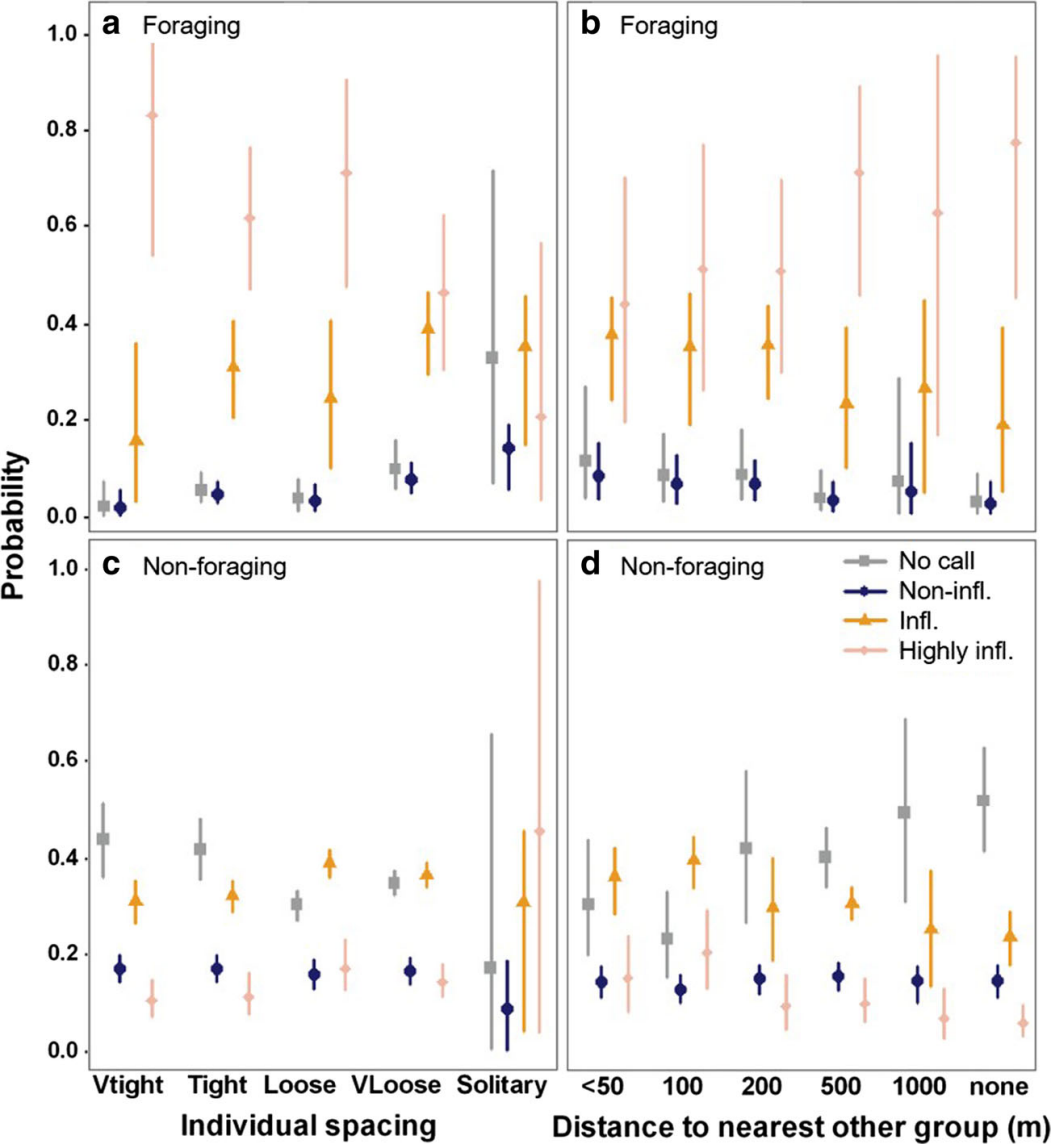
Multivariate GEE model predictions for the significant relationships between the highest level of frequency inflections of calls and group-level behaviour for foraging and non-foraging periods. Levels of frequency inflection: no calls, non-inflected, inflected (1–2 inflections) and highly inflected (> 2 inflections) calls. **a**, **c** Individual spacing. **b**, **d** Distance to nearest other group. Model predictions made to covariate values given in Fig. 3. Infl. = inflected. Note overall higher frequency of inflections during periods of foraging vs. non-foraging periods Error bars represent 95% confidence intervals

The level of call inflections was related to distance between group members, and between groups (Table 3), mainly during foraging periods. In contrast to the high probability of the occurrence of highly inflected calls during foraging periods, during non-foraging periods, inflected calls, or even the absence of calls, were most probable (Fig. 6). During foraging, the probability of occurrence of highly inflected calls increased when group members were more tightly spaced, and decreased at increasing distances between groups (Fig. 6a, b), patterns that were absent during non-foraging periods (Fig. 6c, d). During non-foraging periods, absence of calls became more probable as groups were spaced further apart (Fig. 6d). The wide confidence intervals for the model predictions for the spacing category ‘solitary’ reflect the limited number of observations of solitary individuals (Fig. 6a, c).

## Discussion

We demonstrated that in socially foraging long-finned pilot whales, patterns of social calling varied between periods of foraging and non-foraging behaviour. Moreover, in the presence of larger numbers of individuals, vocal activity (calling and echolocating) was reduced. Pilot whales were relatively silent during non-foraging behaviour and increased their vocal activity during foraging periods of the tagged whale. This occurred not only for echolocation signals, used to locate prey during foraging, but also for calls, most likely serving a communicative role (Jensen et al. 2011; Sayigh et al. 2013; Zwamborn and Whitehead 2017). During foraging periods, the occurrence of highly inflected calls increased during the transition periods directly preceding and following foraging dives, while maintaining high rates during the early descent and late ascent phases of these foraging dives.

### Vocal foragers

Periods of foraging behaviour were characterised by more vocal activity, with more echolocation clicks as well as calls, than during non-foraging periods. These results are consistent with the use of biosonar during foraging dives by deep-diving toothed whales (Miller et al. 2004; Aguilar de Soto et al. 2008; Madsen et al. 2013). Furthermore, the increased call rates suggest that the animals communicate more during foraging periods, when individuals separate from surface group members to dive, than during non-foraging periods.

Highly inflected calls, which were relatively rare overall, were predominantly associated with the early descent phase and late part of the ascent phase of foraging dives, and the near surface periods directly preceding and following these dives. Whereas highly inflected calls were nearly absent at diving depths beyond 350 m of the four tagged whales that dove to these depths, they predominantly occurred during or close in time to foraging dives deeper than 120 m (121–615 m) and were to a lesser extent associated to shallower foraging dives (40–73 m) within the foraging periods. As foraging periods contained foraging dives ranging from 34 to 615 m in depth, this may also reflect the presence of different dive types with associated differences in foraging strategy (such as exploratory foraging or targeting of specific dive depths for different prey), social cohesion and communication.

The increased vocal activity (calling and echolocating) and the use of calls with multiple inflections during foraging may reflect a higher arousal state of the animals, which may be related to the intrinsic changes in behavioural state, group splitting or merging events, or the anticipated or experienced feeding conditions (e.g. King and Janik 2015). Independent of the cause, the context-dependent variation in rate and type of vocalisations could play a role in communication. Since the long-finned pilot whale vocal repertoire may contain distinct call types (Zwamborn and Whitehead 2017; Sayigh et al. 2013 and Marrero Pérez et al. 2017 for short-finned pilot whales), calls with more inflections could represent specific call types produced mainly in a foraging context, or contain other specific spectral, temporal or biphonic characteristics that carry information, next to the number of frequency inflections used to classify calls here. While the overall occurrence of repeated call types has been linked to more social contexts, and is suggested to function to maintain cohesion or contact (Zwamborn and Whitehead 2017), it remains unclear which characteristics of the pilot whale calls function to transfer their information content. Empirical evidence for whether communication takes place should come from observations on call activity or call type-dependent behavioural responses in animals that hear the calls. Conclusive evidence for vocal communication and call-type usage could come from playback experiments of call variants where the actual context variation is absent (e.g. Kate and Jones 1997; Ramos-Fernandéz 2005). These future studies could also include the importance and role of faint calls, excluded from this study due to our focus on calls likely produced by nearby focal group members. Pilot whales disperse more during foraging, both vertically and horizontally, and may decrease their call output levels at depth (Jensen et al. 2011) potentially reducing the overall received level of calls with potential information content for a foraging pilot whale.

The context-dependent use of vocal variants may also be driven by physical conditions for sound production that change with diving depth. Whale calls can be affected by pressure-induced restrictions. Calls produced at depth have a tendency to be shorter and less inflected. They may also contain less energy than calls produced at more shallow depths, and be replaced by communicative echolocation calls (Ridgway et al. 2001; Jensen et al. 2011; Marrero Pérez et al. 2017). Such pressure-related limitations may therefore explain the near-absence of the longer calls with multiple inflections at diving depths > 350 m, while calls with no or few inflections were recorded consistently throughout the depth spectrum and dive cycle. However, this does not preclude that changes in communication needs throughout the dives may also drive changes in the occurrence of different call types.

If the vocal variation reported here plays a role in communication, the information may concern the location as well as intention or motivation of conspecifics. The pilot whales that hear the change in calling rate and type may also recognise group members or specific individuals, as evidence for recognition of vocal identity is widespread among toothed whales (e.g. Ford and Fisher 1983; Janik and Slater 1998; Antunes et al. 2011; Sayigh et al. 2013). In pilot whales, such information would help individuals to relocate their social group and re-establish bonds, for example as they return from foraging dives. More advanced referential signalling about location, quality or abundance of food, using longer and potentially more complex signals that may contain more information, may be a less parsimonious explanation but cannot be excluded (c.f. reports in terrestrial animals: e.g. Templeton et al. 2005; Clay et al. 2012). This is further supported by the increase in call rates and probability of occurrence of highly inflected calls when group members were more tightly spaced at the surface, a combination of vocal and behavioural patterns only observed during foraging periods.

As suggested by Visser et al. (2014), the synchrony in foraging periods (but not necessarily individual foraging dives) of long-finned pilot whales may be related to the signalling of good feeding opportunities by individuals with specific local knowledge. Whether intentional or unintentional, such information is expected to be beneficial in environments where resource distribution is more unpredictable or heterogeneous (Sueur et al. 2011). Indeed, social signalling of food conditions could yield important selective advantages through effects on foraging efficiency. Pilot whales perform energetically demanding dives to reach potential food patches, which they may not be able to fully assess from the surface. Hence, transfer of information on feeding conditions between related, long-term associated group members could provide inclusive fitness benefits for foraging individuals. Social signalling of food conditions to affiliates was also recently suggested for bottlenose dolphins (*Tursiops truncatus*), which produced social signals (calls) associated with food-specific vocalisations (bray calls; King and Janik 2015).

### Silent crowds

Periods of silence in vocal and social animals can occur for several reasons and may even have communicative potential. The absence of vocalisations may for example indicate that the behavioural or social factor driving the production of calls has (temporarily) become absent. Silence was recorded significantly more often during non-foraging periods than during foraging, when the whales rely on sound to find prey. Moreover, during non-foraging periods, whales were more silent when they were in larger, more tightly spaced groups, which is consistent with a functional relief from mediating group cohesion (Palombit 1992; Bradbury and Vehrenkamp 1998; Tyack 2000). Given that a larger number of animals at closer range will increase the likelihood of the recording of calls, the opposite, increased probability of silent periods strengthens the conclusion that the pilot whales were more silent when organised in closely spaced groups at the surface. Previous findings indicated that long-finned pilot whales produce more calls, and a higher proportion of frequency-inflected calls at a larger spread of the aggregation and during more complex behaviours (Taruski 1979; Weilgart and Whitehead 1990). This is in line with our current finding on silent crowds, for which we have surface data on proximity of relatively large numbers of animals, but also confirmation of the absence of deep-diving foraging activity due to the presence of tag data. Long-finned pilot whales off Nova Scotia produced more repeated call sequences in larger groups and while socialising, and less during resting (Zwamborn and Whitehead 2017). These sequences were suggested to function in maintaining social cohesion. The non-foraging state of the pilot whales in our study typically involved resting and or travelling states, while socialising (as predominant group activity) was rarely observed. A silent state may therefore represent a restful or traveling behaviour, where individuals in closer cohesion (able to visually locate associates) and/or at predictable or slow movement patterns can reduce the use of calls aimed to maintain cohesion or contact.

Another reason for silent periods in vocal and social animals may be predation risk. Animals may passively listen for cues of apparent danger and engage in cryptic behaviour to avoid acoustic detection by predators. However, while cetaceans are known to use this silencing strategy (Aguilar de Soto et al. 2011; Rankin et al. 2012), this is likely not the case for pilot whales. When the long-finned pilot whale groups of this study were exposed to playbacks of calls of their potential predators (or food-competitors), killer whales (*Orcinus orca*), they did not respond with cryptic behaviour, but actively approached the sound source while becoming more vocal (Curé et al. 2012). Moreover, given the overall high degree of vocal activity, it seems unlikely that silent periods at the scale of minutes would strongly reduce their acoustic detectability.

### Non-inflected call use

Calls with no or few inflections were present in the acoustic scene of the tagged whale throughout the behavioural spectrum, without a specific association to a social or individual behavioural context examined here. This may mean that these calls convey a less specific message than those with multiple inflections, for example serving a more general purpose of staying in touch with conspecifics and recognition of group members or specific individuals. A study on closely related short-finned pilot whales (*Globicephala macrorhynchus*), thought to have a social organisation and foraging strategy comparable to long-finned pilot whales, also reported relatively short calls without inflections that were suggested to serve in maintaining or re-establishing contact between group members (Jensen et al. 2011), with evidence for distinct diving-related patterns for different call variants in this species (Marrero-Pérez et al. 2017).

## Conclusions

An explicit role for vocal communication in mediating spacing between group members or synchronisation of foraging activity appears plausible. Within the constraints of a limited sample size, elevated calling activity and the use of specific vocalisations with multiple inflections were associated with periods during (descent and ascent) and directly preceding and following foraging dives. In contrast, during non-foraging periods (travel, rest), relatively large and close groups of shallow diving animals limited their vocal activity (calling and echolocating) and turned into silent crowds. These data show that the combination of tag data on vocalisation rate and type, pressure-deduced diving patterns and social surface observations can provide novel insights that may be critical for our understanding of the communication of social toothed whales.

## Electronic supplementary materials

ESM 1 (PDF 590 KB)
