# Supplementary material for: Vocal foragers and silent crowds: context-dependent vocal variation in Northeast Atlantic long-finned pilot whales
Source: Behav Ecol Sociobiol. 2017 Nov 6;71(12):170. doi: 10.1007/s00265-017-2397-y (PMC5674111; doi:10.1007/s00265-017-2397-y)
Supplement: Supplementary file 1 — (PDF 590 KB) [file 265_2017_2397_MOESM1_ESM.pdf]

Electronic supplementary material for the manuscript: 'Vocal foragers and silent crowds: context-dependent vocal variation in Northeast Atlantic long-finned pilot whales', in Behavioral Ecology and Sociobiology, by Fleur Visser, Annebelle CM Kok, Machiel G Oudejans, Lindesay AS Scott-Hayward, Stacy L DeRuiterd, Ana C Alves, Ricardo N Antunes, Saana Isojunno, Graham J Pierce, Hans Slabbekoorn, Jef Huisman and Patrick JO Miller.

Corresponding author: Fleur Visser, Leiden University and Kelp Marine Research, the Netherlands.

Supplementary Figures

Supplementary Figure S1. Examples of call types. a-c: non-inflected calls, d-f: inflected calls; g-i: highly inflected calls.

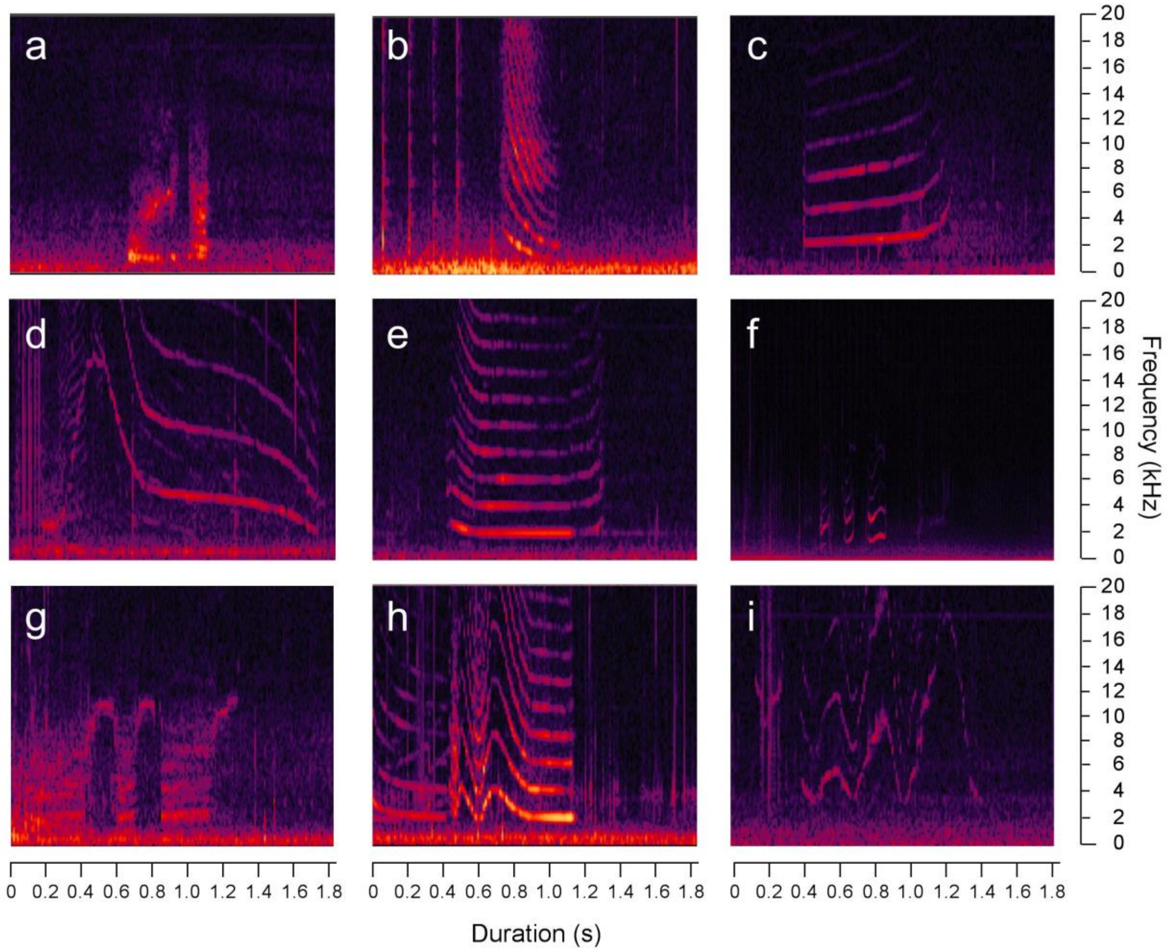

Supplementary Figure S2. Timing in the dive cycle of highly inflected calls produced 0-15 minutes prior to or post deep dives (>34 m), or in the descent, bottom or ascent phase of deep dives, as a function of maximum dive depth of the dive in/closest in time to which they occurred. Open circles: individual calls. Red lines and error bars: mean and SEM.

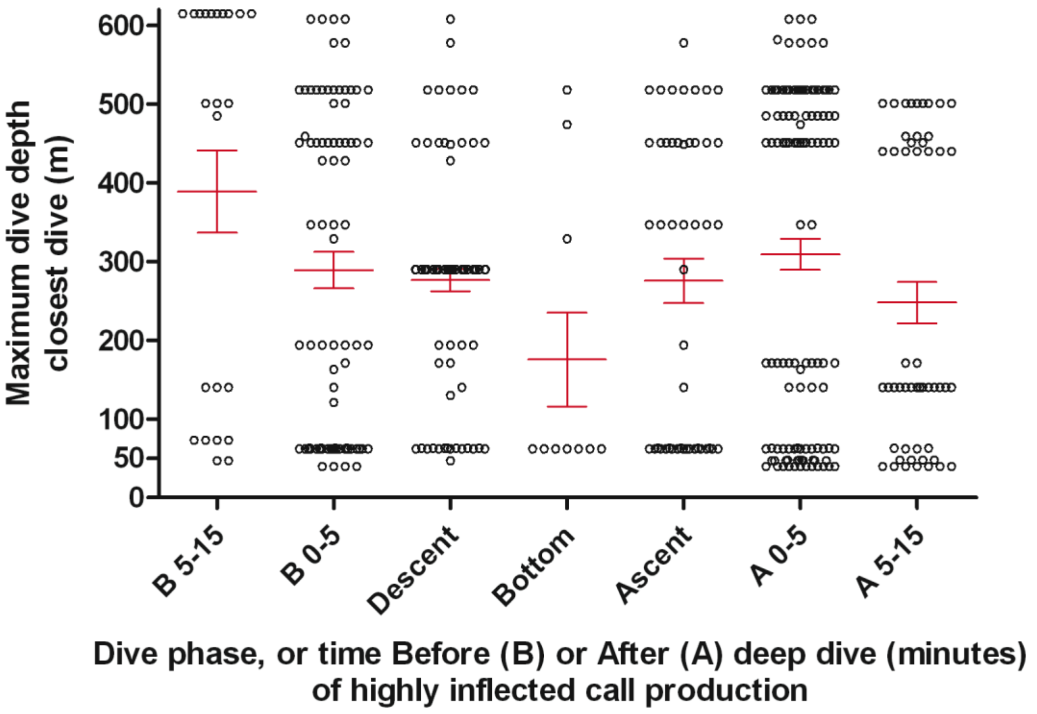

Supplementary Tables

Supplementary Table S1. Parameter estimates and standard errors (±) for retained covariates (*P* < 0.05) in the two binomial GEE-based vocalisation models (silence, calls). Empty cell: parameter not retained best model. Coefficients represent the difference between the factor level and the reference factor level on the scale of the link function (logit).

	Vocalisation type	
Covariate(_factor level)	Silence	Calls
Diving state_shallow	3.7±1.21	-1.8±0.8
Group size	-0.06±0.04[a]	
No. individuals in focal area	0.1±0.01[a]	
Individual spacing_tight	2.4±1.3	-0.7±0.3
Individual spacing_loose	2.1±1.0	-1.2±0.5
Individual spacing_veryLoose	2.5±1.2	-0.6±0.4
Individual spacing_solitary	2.0±1.3	-2.2±0.3
Diving state_shallow:Group size	0.22±0.02	
Diving state_shallow:No. individuals in focal area	-0.1±0.02	
Diving state_shallow:Individual spacing_tight	-2.5±1.3	1.1±0.7
Diving state_shallow:Individual spacing_loose	-3.1±0.9	1.7±0.8
Diving state_shallow:Individual spacing_veryloose	-3.5±1.2	1.0±0.9
Diving state_shallow:Individual spacing_solitary	-3.6±2.2	3.9±0.9

[a]Parameter retained in best model because included in significant 2-way interaction term

Supplementary Table S2. Parameter estimates and standard errors (±) for retained covariates ($P < 0.05$) in the multinomial GEE-based vocalisation model (responses: no calls, non-inflected, inflected or highly inflected calls). Coefficients represent the difference between the factor level and the reference factor level on the scale of the link function (cumulative logit).

Covariate(_factor level)	Call modularity
Intercept 1 (no calls)	0.16±0.25
Intercept 2 (non-inflected calls)	0.84±0.22
Intercept 3 (inflected calls)	2.63±0.24
Diving state_deep	-3.82±0.62
Distance to nearest other group_100to200	-0.40±0.47[a]
Distance to nearest other group_10to50	-0.89±0.34[a]
Distance to nearest other group_200to500	-0.47±0.20[a]
Distance to nearest other group_500to1000	-0.10±0.50[a]
Distance to nearest other group_50to100	-1.24±0.22[a]
Individual spacing_loose	-0.56±0.19
Individual spacing_solitary	-1.83±1.51
Individual spacing_tight	-0.07±0.11
Individual spacing_veryLoose	-0.36±0.16
Diving state_deep:Distance to nearest other group_100to200	1.68±0.38
Diving state_deep:Distance to nearest other group_10to50	2.46±0.23
Diving state_deep:Distance to nearest other group_200to500	0.84±0.29
Diving state_deep:Distance to nearest other group_500to1000	0.80±1.02
Diving state_deep:Distance to nearest other group_50to100	2.49±0.97
Diving state_deep:Individual spacing_loose	1.33±0.34
Diving state_deep:Individual spacing_solitary	5.11±1.02
Diving state_deep:Individual spacing_tight	1.31±0.52
Diving state_deep:Individual spacing_veryLoose	2.22±0.67

[a]Parameter retained in best model because included in significant 2-way interaction term.
